# Antiretroviral Therapy Changes for Medicare Beneficiaries With HIV Transitioning to Long-Term Care

**DOI:** 10.1001/jamanetworkopen.2025.48936

**Published:** 2025-12-12

**Authors:** Brianne Olivieri-Mui, Ira B. Wilson, Ellen P. McCarthy, Laiji Yang, Mark Brennan Ing, Laura Senier, Dae Hyun Kim

**Affiliations:** 1 Department of Public Health and Health Sciences, Northeastern University, Boston, Massachusetts; 2 The Marcus Institute for Aging Research, Hebrew SeniorLife, Harvard Medical School, Boston, Massachusetts; 3 Department of Health Services, Policy and Practice, Brown University, Providence, Rhode Island; 4 Brookdale Center for Healthy Aging, Hunter College, The City University of New York, New York, New York; 5 Department of Sociology & Anthropology, Northeastern University, Boston, Massachusetts

## Abstract

**Importance:**

Studies of nursing home (NH) residents show lower than expected antiretroviral therapy (ART) use, but it is unclear whether ART use changes across the transition from the community to long-term NH stay.

**Objective:**

To examine changes in ART use across the transition from the community to long-term NH stay.

**Design, Setting, and Participants:**

This retrospective cohort study examined long stays in US NHs for people with HIV in a sample of 5% of Medicare claims from 2014 to 2019. Stays were at least 30 days long, had at least 3 months between multiple stays, and were for those continuously enrolled in Medicare for the stay and 6 months before. Analysis was completed in May 2025.

**Exposures:**

Admission year demographics included age, race and ethnicity (non-Hispanic Black, non-Hispanic White, and other [American Indian or Alaska Native, Asian or Pacific Islander, other, and unknown]), binary sex, Medicaid eligibility, whether the stay was preceded by a skilled nursing stay, and whether disability was Medicare original eligibility. NH characteristics included for-profit status, census region, and facility quality rating.

**Main Outcomes and Measures:**

Linear regression estimated changes in the proportion of days covered by 3-drug ART, and hierarchical multinomial logistic regression estimated the risk of never having, losing, or gaining ART vs always having it, across the transition from the community to long-term NH stay.

**Results:**

There were 713 long NH stays for 657 people with HIV (mean [SD] age, 61.0 [11.4] years) across 598 facilities; 271 stays (38%) were for people aged 65 years and older. Only 23 individuals lost ART (3%), 97 individuals (14%) gained ART, 185 individuals (26%) never had ART, and 408 individuals (57%) always had ART across the transition. Excluding those who lost ART, all other groups were mostly men (never, 132 men [71%]; always, 289 men [71%]; gained, 72 men [74%]) and Black (never, 85 individuals [46%]; always, 237 individuals [58%]; gained, 58 individuals [60%]). There was an increase in the proportion of days covered (mean intercept α = 13.92; 95% CI, 9.57-18.29). Compared with always having ART, Black race (relative risk [RR], 0.52; 95% CI, 0.35-0.77), polypharmacy (RR, 0.41; 95% CI, 0.23-0.74), and disability as original Medicare eligibility (RR, 0.47; 95% CI, 0.29-0.77) were associated with lower risk of never having ART. For-profit facilities were associated with higher risk (RR, 1.63; 95% CI, 1.03-2.59) of never having ART. Polypharmacy was associated with lower risk of gaining ART (RR, 0.15; 95% CI, 0.05-0.49).

**Conclusions and Relevance:**

These findings suggest that long-term NH stays may be associated with improved ART use among people with HIV because most stays without ART never had ART before admission.

## Key Points

**Question:**

How do the days covered by antiretroviral therapy prescriptions change when Medicare beneficiaries with HIV transition from the community to a long nursing home stay?

**Findings:**

In this cohort study of 713 long stays for 657 individuals with HIV across 598 facilities, the proportion of days covered by antiretroviral therapy prescriptions increased by a mean intercept (α value) of 13.92 after transitioning to a nursing home. More than one-quarter of nursing home stays were without prescriptions for antiretroviral therapy before and after nursing home admission.

**Meaning:**

These findings suggest that nursing homes can maintain or even improve existing engagement with antiretroviral therapy for people with HIV but still miss opportunities for treatment initiation.

## Teaser

This cohort study examines changes in antiretroviral use among Medicare-enrolled individuals with HIV across the transition from living in the community to long-term nursing home stays.

## Introduction

Nursing homes (NHs) play an increasingly important role in clinical care for people with HIV, reflected in the doubling of long NH stays that are made up by this population since 2001.^1^ This upward trend is likely to continue because people with HIV are reaching older ages than earlier in the epidemic and are more likely to have fewer social and financial supports that otherwise facilitate aging at home, compared with people without HIV.^2,3,4^ Moreover, more than one-half of the 1.2 million people with HIV in the US are older than 50 years, displaying signs and symptoms of aging equivalent to those of people with more advanced age but without HIV.^4,5^ As such, it is important that we understand the role NHs play in maintaining good health among people with HIV, which is only possible with the use of antiretroviral therapy (ART).

Evidence shows that care quality for HIV in the NH setting may be lower than expected.^6,7,8^ The majority of people with HIV using NHs are using facilities in the Southern census region, where there is higher HIV prevalence than other parts of the US.^9^ Moreover, up to 90% of people with HIV using NHs are dually eligible for Medicaid, an indicator of low income.^7^ In the context of what is known about the process of selecting an NH, it is often based on proximity to a person’s home or family, and choices are limited. As a result, people with HIV more often use lower quality NHs.^8,10^

Current research of Medicare data consistently shows that 20% to 25% of people with HIV in NHs have no ART; in a recent review of Medicaid programs, that number was as high as 85% in some states.^9,11^ Continuation of ART during an NH stay is the responsibility of the facility. However, research among people with HIV who are already NH residents has shown that NHs have varying degrees of compliance with ART guidelines.^6,7^ As of 2013, ART is recommended for all people with HIV because it is key to viral suppression and is the most basic form of HIV care and prevention.^12^ Research has also demonstrated that the longer a person with HIV is in an NH the more likely they are to have ART^13^ and that NHs with higher proportions of people with HIV as residents have better health outcomes than those with less experience with HIV.^8^ Compared with the community setting, where older age is often associated with levels of adherence ranging from 80% to 90%,^14,15^ the evidence implies a reduction in ART use while transitioning from the community to a long NH stay. However, previous research has been limited by selection bias, where the study includes either people with HIV who are only outside the NH setting or people with HIV who are only residents of an NH setting, which precludes understanding changes across the transition.

Therefore, this study builds on the previous literature by bridging what we know about ART use in the community with what happens after becoming a long-stay NH resident. The goal of this study was to estimate the change in ART use across the transition from community living to a long NH stay among a Medicare population with HIV in NHs in the US. We hypothesized that, on average, people with HIV would have reduced ART adherence after entering an NH and that lower quality NHs would have greater reductions in ART adherence.

## Methods

### Data

In this retrospective cohort study, we used longitudinal data from a 5% random sample of 2014 to 2019 traditional Medicare beneficiaries from the Centers for Medicare & Medicaid Services that included the Chronic Conditions Data Warehouse (CCW) files, which capture the first date of diagnosis for 67 chronic and other disabling conditions.^16,17,18^ The Master Beneficiary Summary File provided demographic and Medicaid and Medicare enrollment information. Medicare Part D prescription claims data were used to calculate polypharmacy and ART use, as described below. This study was approved by the Northeastern University institutional review board and Advara institutional review board for The Marcus Institute for Aging Research, Hebrew SeniorLife, which approved a waiver of informed consent. Reporting of the results adhered to the Strengthening the Reporting of Observational Studies in Epidemiology (STROBE) reporting guidelines.^19^

### Population

We studied long NH stays among people with HIV. Before identifying stays, people with HIV were identified as beneficiaries with at least 1 HIV diagnosis in Part A (inpatient claims file) or 2 diagnoses at least 1 week apart in Part B (outpatient carrier file), or who had at least 2 successive fills for an antiretroviral medication (excluding lamivudine, which can be used to treat hepatitis) in Part D from 2014 to 2019.^13,20,21^ Long stays were derived from a validated claims-based algorithm (sensitivity, 0.88; specificity, 0.93) as the first date of NH residence not billed as a Medicare Part A skilled nursing facility (SNF).^22^ This approach captured long-term stays that began after an SNF stay subsequent to a qualifying hospitalization—if one existed—or that began directly from the community. We excluded stays with less than 3 months of community time before admission to the earlier of a hospitalization, SNF stay, or long-stay admission. The long NH stay was at least 30 days^23^ and was associated with a person with HIV eligible for Medicare 6 months preceding long-stay admission and for the duration of the stay of interest. Stays had to occur after the first evidence of HIV in the claims (Figure).

**Figure.**
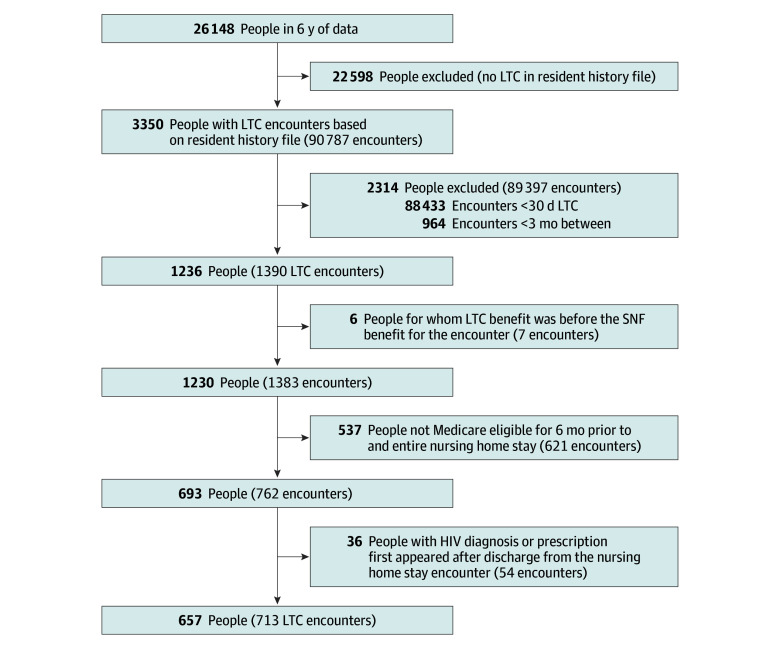
Enrollment Flowchart for People With HIV and Long-Term Care (LTC) Stays Included in the Study SNF indicates skilled nursing facility.

### Outcomes

Change in ART use was calculated as a function of implementation based on the proportion of days covered by a prescription for an ART regimen—defined as at least 3 concurrent antiretroviral medications—at 2 different time periods: (1) the last 90 days in the community and (2) up to the first 90 days after long-stay admission if there was no death, Medicare disenrollment, or end of the data, December 31, 2019; period 2 minus period 1 made up the primary outcome, continuous change in ART use (eFigure in Supplement 1). The secondary outcome, change in ART use as a categorical variable, was based on ever having access to any ART regimen in period 1 and 2, which created 4 groups of ART change: never had ART in period 1 or 2, always had ART, gained ART, and lost ART.

### Demographics and Covariates

The proportion of days covered in period 1 and 2 was also used to created 3 categories estimating ART adherence, for descriptive purposes: no ART (had no claims for ART), ART nonadherent (<80% proportion of days covered), and ART adherent (≥80% proportion of days covered).^24^ The Master Beneficiary Summary File for the year of the long stay provided age (centered for regression analyses), race and ethnicity (Centers for Medicare & Medicaid services variable RTI_RACE_CD aggregated as non-Hispanic Black, non-Hispanic White, and other [American Indian or Alaska Native, Asian or Pacific Islander, other, and unknown]), sex (male or female), date of death, US census region (Northeast, South, Midwest, and West [including Hawaii and Alaska]), and whether the beneficiary was dually eligible for Medicare and Medicaid (ever in the admission year [dually eligible]). Data on race and ethnicity are included because HIV disproportionately affects Black and Hispanic populations. Latent health profiles for comorbidities categorized people according to the dominant morbidity, as described elsewhere (see eMethods in Supplement 1 for details).^25^ Three health profiles emerged: substance use, cardiovascular or pulmonary, and multisystem (which had several prevalent deficits). A validated Medicare claims–based frailty index was calculated on the basis of claims during the 6 months prior to long-stay admission and classified beneficiaries as not frail (<0.25) or frail (≥0.25) at each stay.^26,27,28^ Polypharmacy was classified as having 5 or more nonantiretroviral anatomic therapeutic class third tier (Anatomical Therapeutic Chemical classification 3) medications concurrently in the 90 days prior to the earlier of hospital, SNF, or long-stay admission.

### Statistical Analysis

Chi-squared tests and analysis of variance were used to assess differences in the distribution of categorical and continuous variables, respectively, across change groups. We used hierarchical linear and multinomial logistic regression models, with robust estimators to account for nesting within NHs and people, to estimate the association of person and facility factors with changes in ART use as a continuous variable and categorical variable, respectively. All models were adjusted for variables significant at the *P* < .05 level in univariate regression, to achieve parsimony; power analysis is shown in the eMethods in Supplement 1. Analyses were completed in May 2025, used 2-sided hypothesis tests at α = .05, and used SAS statistical software version 9.4 (SAS Institute).

## Results

### Descriptive Characteristics

In this US Medicare sample, there were 713 long stays for 657 people with HIV across 598 NHs (Figure). The eTable in Supplement 1 summarizes the included and excluded people with HIV; 271 stays (38%) were for people aged 65 years and older. Excluding those who lost ART, all other groups were mostly men (never, 132 men [71%]; always, 289 men [71%]; gained, 72 men [74%]). The mean (SD) age for people with HIV who had long NH stays was 61.0 (11.4) years. After transitioning to the NH, the proportion of days covered by ART improved (adjusted mean intercept, α = 13.92; 95% CI, 9.57-18.29).

Table 1 summarizes the 4 distinct categories of ART change. The always ART group was the largest, with 408 individuals (57%); 97 individuals (14%) gained ART, 185 individuals (26%) never had ART, and 23 individuals lost ART (3%). Across the ART change groups, the lost ART group was the youngest at a mean (SD) age of 59.7 (11.4) years, whereas the never ART group was the oldest with a mean (SD) age of 63.5 (13.7) years. All ART groups were predominantly Black (never ART, 85 individuals [46%]; always ART, 237 individuals [58%]; gained ART, 58 individuals [60%]), dually eligible for Medicaid (never ART, 99 individuals [53%]; always ART, 243 individuals [60%]; gained ART, 65 individuals [67%]), and frail (never ART, 144 individuals [78%]; always ART, 283 individuals [69%]; gained ART, 72 individuals [74%]). Polypharmacy was most prevalent among the always ART group (77 individuals [19%]). The cardiovascular and pulmonary comorbidity profile was the most prevalent (never ART, 95 individuals [51%]; always ART, 200 individuals [49%]; gained ART, 44 individuals [45%]; lost ART group is too small to report) in all groups. The proportion of stays where the people with HIV were originally disability eligible was higher in the always ART (327 individuals [80%]) group than the never ART (120 individuals [65%]) or gained ART (71 individuals [73%]) groups. The never ART group had the highest proportion who died within a year of admission (60 individuals [32%]). Sample sizes smaller than the minimum reportable value (11 individuals) preclude reporting values for several characteristics, particularly for the lost ART group.

**Table 1.** Demographic Characteristics of Each ART Change Group^a^

Characteristic	Participants or NHs, No. (%)				*P* value
	Never ART (n = 185)	Always ART (n = 408)	Lost ART (n = 23)^b^	Gained ART (n = 97)	
Participant characteristics					
Age, mean (SD), y	63.54 (13.69)	60.35 (10.15)	59.71 (11.43)	60.06 (10.99)	.05
Sex					
Male	132 (71)	289 (71)	NA	72 (74)	.32
Female	53 (29)	119 (29)	NA	25 (26)	
Race and ethnicity					
Non-Hispanic Black	85 (46)	237 (58)	NA	58 (60)	.04
Non-Hispanic White	85 (46)	128 (31)	NA	NA	
Other^c^	15 (8)	43 (10)	NA	NA	
Dually eligible^d^	99 (54)	243 (60)	NA	65 (67)	.15
Frailty group					
Not frail	41 (22)	125 (31)	NA	25 (26)	.13
Frail	144 (78)	283 (69)	NA	72 (74)	
Polypharmacy	17 (9)	77 (19)	NA	NA	<.001
Proportion of days covered, %					
Before entering NH					
Mean (SD)	0	72.92 (27.80)	52.03 (33.71)	0	NA
Median (IQR)	0	81.11 (54.44-98.88)	34.44 (26.66-96.66)	0	NA
After entering NH					
Mean (SD)	0	82.91 (22.29)	0	71.85 (25.35)	NA
Median (IQR)	0	92.22 (76.66-98.88)	0	80.00 (46.66-94.44)	NA
Comorbidity latent profile					
Substance use	67 (36)	142 (35)	NA	40 (41)	.72
Cardiovascular or pulmonary	95 (51)	200 (49)	NA	44 (45)	
Multisystem deficits	23 (12)	66 (16)	NA	13 (13)	
Disability original eligibility^e^	120 (65)	327 (80)	NA	71 (73)	<.001
Died within 1 y^f^	60 (32)	76 (19)	NA	17 (17)	<.001
NH characteristics					
Census region					
Northeast	43 (23)	126 (31)	NA	21 (22)	.13
South	78 (42)	144 (35)	NA	40 (41)	
West	19 (10)	56 (14)	NA	12 (12)	
Midwest	45 (24)	82 (20)	NA	24 (25)	
Certified beds, No.					
Mean (SD)^g^	136 (73)	154 (132)	142 (81)	156 (119)	.33
Median (IQR)	120 (94-161)	120 (93-157)	120 (85-160)	120 (102-180)	
Total residents, No.					
Mean (SD)	115 (67)	131 (119)	117 (75)	133 (117)	.31
Median (IQR)	105 (77-131)	104 (75-135)	107 (73-138)	103 (82-133)	
For-profit status	150 (81)	290 (71)	NA	73 (75)	.08
Star rating					
1	36 (20)	62 (15)	NA	20 (21)	<.001
2	45 (24)	98 (24)	NA	23 (24)	
3	39 (21)	64 (16)	NA	18 (19)	
4	35 (19)	81 (20)	NA	15 (15)	
5	29 (16)	100 (25)	NA	17 (17)	

Abbreviations: ART, antiretroviral therapy; NA, not applicable; NH, nursing home.

^a^ ART change groups are determined by comparing the time before admission to the time after becoming a long-stay NH resident. Groups include never had ART, always had ART, lost ART after transitioning to long-stay NH, and gained ART after transitioning to long-stay NH.

^b^ Most of the data in the lost ART group are redacted because of small sample sizes. Medicare values less than 11 cannot be reported.

^c^ Other refers to American Indian or Alaska Native, Asian or Pacific Islander, other, and unknown.

^d^ Refers to dual eligibility for Medicaid and Medicare.

^e^ Originally eligible for Medicare due to disability.

^f^ Refers to average days until death for only those who died (267 individuals [37%]).

^g^ Refers to number of Medicare or Medicaid certified beds in the facility.

Stays were slightly more often in the south (never ART, 78 stays [42%]; always ART, 144 stays [35%]; gained ART, 40 stays [41%]). Most stays took place in for-profit facilities (never ART, 150 stays [81%]; always ART, 290 stays [71%]; gained ART, 73 stays [75%]). A plurality of stays in the gained ART group were at 1-star (20 stays [21%]) or 2-star (23 stays [24%]) facilities, compared with 4-star (81 stays [20%]) or 5-star (100 stays [25%]) facilities for the always ART group and 2-star (45 stays [24%]) or 3-star (39 stays [21%]) facilities for the never ART group.

### Mean Changes in Proportion of Days Covered From Linear Regression

Few variables were significantly associated with the continuous variable for change in ART proportion of days covered; age, polypharmacy, and dual eligibility for Medicare and Medicaid remained in the model. Overall, these data estimated an adjusted mean increase of 13.92 (95% CI, 9.57 to 18.29) in proportion of days covered across the transition from the community to long-term NH stay for people with HIV when mean age was 61 years, there was no polypharmacy, and people with HIV were not dually eligible for Medicaid (Table 2). Polypharmacy was associated with a negative change in mean proportion of days covered (b = −25.01; 95% CI, −31.34 to −18.68), and dual eligibility was associated with a mean increase in proportion of days covered (b = 5.78; 95% CI, 0.23 to 11.33).

**Table 2.** Linear Regression for Continuous Change in the PDC by ART^a^

Variable	Mean change in PDC, β (95% CI)
Intercept	13.92 (9.57 to 18.29)
Age	−0.20 (−0.42 to 0.02)
Polypharmacy	−25.01 (−31.34 to −18.68)
Dually eligible	5.78 (0.23 to 11.33)

Abbreviations: ART, antiretroviral therapy; PDC, proportion of days covered.

^a^ Relative mean change in PDC equals the β coefficients from multilinear regression adjusting for only age (centered on the mean age of 61 years), polypharmacy, and dual eligibility for Medicare and Medicaid (dually eligible). Change in ART was calculated as the proportion of days with 3-drug ART after long-stay admission minus the proportion of days with a 3-drug ART regimen in the last 3-months in the community before long-stay admission. Therefore, negative numbers indicate that the adherence after long-stay admission was lower than adherence in the community.

### Associations With ART Change Groups From Multinomial Logistic Regression

Multinomial logistic regression for the ART groups (with the always ART group as the reference), showed that Black race compared with White race (relative risk [RR], 0.52; 95% CI, 0.35-0.77), disability original eligibility (RR, 0.47; 95% CI, 0.29-0.77), and polypharmacy (RR, 0.41; 95% CI, 0.23-0.74) were associated with lower risk of never having ART, but for-profit NH status was associated with higher risk (RR, 1.63; 95% CI, 1.03-2.59) of never having ART (Table 3). Polypharmacy was associated with lower risk of being in the gained ART group (RR, 0.15; 95% CI, 0.05-0.49), compared with the always ART group. The lost ART group was not different from the always ART group according to these data.

**Table 3.** Multinomial Logistic Regression Estimates for the ART Change Group

Variables	aRR (95% CI)			
	Never ART^a^	Lost ART	Gained ART	Always ART
Age	1.01 (0.99-1.03)	0.99 (0.94-1.04)	0.99 (0.97-1.01)	1.00 [Reference]
Race and ethnicity				
Non-Hispanic Black	0.52 (0.35-0.77)	0.89 (0.34-2.29)	0.95 (0.57-1.58)	1.00 [Reference]
Non-Hispanic White (reference)	1.00 (1.00-1.00)	1.00 (1.00-1.00)	1.00 (1.00-1.00)	1.00 [Reference]
Other^b^	0.58 (0.29-1.15)	1.17 (0.28-4.88)	0.73 (0.28-1.91)	1.00 [Reference]
Dual eligible^c^	0.82 (0.56-1.19)	0.75 (0.31-1.8)	1.40 (0.87-2.26)	1.00 [Reference]
Polypharmacy	0.41 (0.23-0.74)	0.43 (0.10-1.91)	0.15 (0.05-0.49)	1.00 [Reference]
Frail	1.41 (0.92-2.16)	2.15 (0.69-6.71)	1.25 (0.72-2.16)	1.00 [Reference]
Disability original eligible^d^	0.47 (0.29-0.77)	1.44 (0.32-6.56)	0.57 (0.30-1.07)	1.00 [Reference]
For profit	1.63 (1.03-2.59)	1.14 (0.38-3.37)	1.17 (0.68-2.00)	1.00 [Reference]
Overall star rating				
2 Stars	0.76 (0.43-1.35)	0.41 (0.07-2.45)	0.72 (0.37-1.41)	[1.00 [Reference]
3 Stars	1.05 (0.58-1.88)	1.60 (0.38-6.69)	0.85 (0.40-1.80)	1.00 [Reference]
4 Stars	0.78 (0.43-1.41)	1.36 (0.32-5.87)	0.60 (0.28-1.28)	1.00 [Reference]
5 Stars	0.60 (0.33-1.08)	1.61 (0.37-6.96)	0.58 (0.29-1.18)	1.00 [Reference]
1 Star (reference)	1.00 (1.00-1.00)	1.00 (1.00-1.00)	1.00 (1.00-1.00)	1.00 [Reference]

Abbreviations: aRR, adjusted relative risk; ART, antiretroviral therapy.

^a^ ART change groups are determined by comparing the time in the last 3 months in the community to the time after long-stay admission. Groups include never had ART, always had ART, lost ART after long-stay admission, and gained ART after long-stay admission.

^b^ Other refers to American Indian or Alaska Native, Asian or Pacific Islander, other, and unknown.

^c^ Refers to being dually eligible for Medicaid and Medicare in the year of the admission.

^d^ Refers to being originally eligible for Medicare because of disability.

## Discussion

This cohort study aimed to understand changes in ART use for Medicare beneficiaries with HIV transitioning from the community to long NH stays. We found that among a group with a mean age of 61 years, ART use seemed to improve after the transition, that there was no ART use before or after the transition for nearly one-quarter of our sample, and that comorbidities and frailty had no association with ART changes. These findings are contrary to our hypothesis that posited lower ART use after the transition and that lower NH quality rating would be associated with even lower ART use. These findings are critically important to our understanding of NH care for people with HIV because they dispel select concerns that the transition to long-stay NH resident and the transition from Medicare Part A to Part D medication benefits are opportunities for reduced ART use.

Few NHs have experience caring for people with HIV, with many seeing only 1 or 2 individuals in a 3-year span.^9^ Experience with HIV care correlates with better health outcomes in both the NH setting and the outpatient setting.^8^ Many community-based studies have found that better adherence to ART is associated with older age,^29,30^ and 1 study of NH residents with HIV showed that longer duration of an NH stay was associated with better ART adherence,^13^ although that same study found that 21% of people with HIV in NHs had no ART. Without following people from the outpatient or community setting into the NH setting, these previous studies were limited in their generalizability because of selection bias; they examined only people with HIV using outpatient services, or only people with HIV using NHs. In our approach, we mitigated that limitation and demonstrated that on average ART use may increase across the transition from the community to long NH stay, but that approximately one-quarter of people with HIV who transition to long-stay NH residents never have ART before or after the transition. Therefore, the previous research showing many people with HIV without ART in the NH setting was more likely reflecting those who never had ART even before admission, rather than any action by an NH to reduce ART use. NHs seem to maintain patient engagement with ART.

The most interesting group we saw in our data was the group never having ART before or after transitioning to long-stay NH resident status, despite that they had diagnosis codes for HIV. This group was older, which is counter to previous reports.^31^ This group also was less often originally eligible for Medicare through disability, more often in for-profit NHs, and made up around one-quarter of the stays studied. Our findings are consistent with research showing approximately 20% to 25% of older people with HIV are out of care either in the community or in the NH.^9,31^ Going a step further, we have confirmed that this phenomenon does not change when tracking people across that transition, as we would hope. This may be the result of challenges in access to HIV-informed or culturally affirming care, as has been reported across the southern US, where many rural areas have high HIV incidence and limited clinical resources.^32^ However, our study implies that despite challenges for community-dwelling people with HIV, there is an opportunity for NHs to identify people with HIV who are out of care by strategically employing innovative HIV or communicable disease testing at admission; this may be an area for future research.

A partial focus of our hypothesis was lower ART use associated with lower quality ratings of NHs. In our analysis, star ratings were not associated with ART use at all. Similarly, factors often associated with worse health outcomes, such as polypharmacy, comorbidities, and frailty, had unexpected results. Polypharmacy in a non-HIV population is often associated with worse health outcomes, where deprescribing interventions are then used to improved adherence.^33,34^ In a population with HIV, studies have shown that polypharmacy is associated with better ART adherence, confirming our finding.^24^ However, comorbidities and frailty, which are often associated with worse health outcomes and lower ART use, had no association with the change in ART.^25,35,36,37^

### Limitations

We strove to ensure internal validity but acknowledge certain limitations to this study. First, enrollment in traditional Medicare before and during an NH stay may limit the generalizability of our findings within and outside of the US. Second, the algorithm to identify people with HIV does not account for people who never sought treatment for their HIV, the duration of HIV infection, or whether they used fewer than 3 antiretrovirals.^9,21^ In addition, comorbidities were accounted for using health profiles based on conditions ever diagnosed by the CCW potentially missing people with HIV outside CCW diagnosis criteria and equally weights all conditions regardless of the number of conditions included in an index. However, validity has been demonstrated using CCW data.^25^ Third, we used an algorithm to establish long-stay start date as the index date and data prior could have occurred during SNF or hospital stays where bundled payments preclude itemized claims that would show changes in active diagnoses or treatment before long-stay admission was achieved but after leaving the community. Fourth, we did not include characteristics of a hospitalization or SNF stay, if they existed before the long-stay admission. Fifth, we used a validated function of implementation^38^ to measure ART adherence at both time points and based on fills in Part D claims, which do not account for treatment history or confirm that a patient ingested medication, and misses use of 2-drug regimens, potentially overestimating the prevalence of suboptimal adherence.^39^

## Conclusions

In this cohort study of older adults with HIV, we showed that access to ART seemingly improved across the transition from the community to long-term NH stay and that those without ART in the NH likely did not have ART before their admission. These findings suggest that NHs can help maintain or even improve existing engagement with ART for people with HIV but opportunities for treatment initiation are still missed.

## Notes

Supplement 1. eMethods. eTable. Characteristics of people with HIV included compared to those excluded (n=657 people with HIV made up n=713 stays for the study) eFigure. Timeline for assessing proportion of days covered (proportion of days covered) before and after long-stay admission which considered if there was a hospital (Hosp) or skilled nursing facility (SNF) stay preceding long-stay admission/first data of long-stay status

Supplement 2. Data Sharing Statement
