# Supplementary material for: Antiretroviral Therapy Changes for Medicare Beneficiaries With HIV Transitioning to Long-Term Care
Source: JAMA Netw Open. 2025 Dec 12;8(12):e2548936. doi: 10.1001/jamanetworkopen.2025.48936 (PMC12701507; doi:10.1001/jamanetworkopen.2025.48936)
Supplement: Supplement 1. — eMethods. eTable. Characteristics of people with HIV included compared to those excluded (n=657 people with HIV made up n=713 stays for the study) eFigure. Timeline for assessing proportion of days covered (proportion of days covered) before and after long-stay admission which considered if there was a hospital (Hosp) or skilled nursing facility (SNF) stay preceding long-stay admission/first data of long-stay status [file jamanetwopen-e2548936-s001.pdf]

Supplemental Online Content

Olivieri-Mui B, Wilson IB, McCarthy EP, et al. Antiretroviral therapy changes for Medicare beneficiaries with HIV transitioning to long-term care. *JAMA Netw Open*. 2025;8(12):e2548936. doi:10.1001/jamanetworkopen.2025.48936

eMethods

eTable. Characteristics of people with HIV included compared to those excluded (n=657 people with HIV made up n=713 stays for the study)

eFigure. Timeline for assessing proportion of days covered (proportion of days covered) before and after long-stay admission which considered if there was a hospital (Hosp) or skilled nursing facility (SNF) stay preceding long-stay admission/first data of long-stay status

This supplemental material has been provided by the authors to give readers additional information about their work.

© 2025 Olivieri-Mui B et al. *JAMA Network Open*.

eMethods:

Power calculation

A priori we estimated 20,160 people with HIV in our data, 2.5% (n=500) with new long NH stays, polypharmacy as a covariate to be 70%, a 2-sided alpha of 0.05, and intra-class-correlation of 0.15 for at least 2 people per NH. Prior research shows average community PDC of 84.5% (standard deviation 18.3%). Therefore we had >80% power to detect a moderate effect size of 0.5 between patients with and without polypharmacy, one of the more common geriatric conditions.

Latent profiles for comorbidities as a covariate

Latent health profiles for comorbidities categorized people based on the dominant morbidity as previously described in the literature.[22] Comorbidities and conditions were present if the CCW diagnosis dates were before the post-acute SNF stay admission date.[38,39] Each diagnosis belonged to one of the eight indices, with the following ranges: mental health (range: 0-6), substance use (0-4), chronic conditions (0-11), cardiovascular conditions (0-9), sensory conditions (0-4), musculoskeletal conditions (0-10), pulmonary conditions (0-2), and learning disabilities/neurologic conditions (0-9). Latent profile analysis, accounting for people with HIV having multiple post-acute SNF stays, was performed using Stata *gsem* (Stata version 15). Health profiles were based on the eight indices, and controlled for age, sex, and race/ethnicity to meet the assumption of conditional independence. We tested the fit of 1 to 6 health profiles and selected the profile arrangement with the lowest Bayesian information criterion (BIC), ensuring that each health profile had a sample size of at least 100. Named based on the indices with the highest median values, three health profiles emerged: substance use, cardiovascular/pulmonary, and multisystem (which had several categories with high median values).

© 2025 Olivieri-Mui B et al. *JAMA Network Open*.

eTable. Characteristics of people with HIV included compared to those excluded (n=657 people with HIV made up n=713 stays for the study)

Characteristic	Study_included	Study_excluded
n	657	25491
Age mid 2014 (mean +/- SD)	59.1+/-11.3	53.9+/-11.6
	n(%)	n(%)
Female	189 (28.8)	6822 (26.8)
Non-Hispanic Black/African American	363 (55.3)	10970 (43.0)
Non-Hispanic White	231 (35.2)	10265 (40.3)
Other race/eth	63 (9.6)	4256 (16.7)
Dually eligible for Medicare/Medicaid	477 (72.6)	14049 (55.1)
Original eligibility for Medicare was disability	489 (74.4)	20468 (80.3)

eFigure. Timeline for assessing proportion of days covered (proportion of days covered) before and after long-stay admission which considered if there was a hospital (Hosp) or skilled nursing facility (SNF) stay preceding long-stay admission/first data of long-stay status.

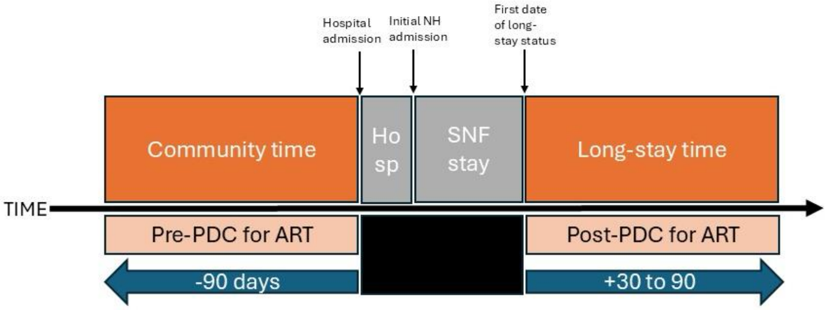

© 2025 Olivieri-Mui B et al. *JAMA Network Open*.
