# Supplementary material for: Antiretroviral Therapy Changes for Medicare Beneficiaries With HIV Transitioning to Long-Term Care
Source: JAMA Netw Open. 2025 Dec 12;8(12):e2548936. doi: 10.1001/jamanetworkopen.2025.48936 (PMC12701507; doi:10.1001/jamanetworkopen.2025.48936)
Supplement: Supplement 2. — Data Sharing Statement [file jamanetwopen-e2548936-s002.pdf]

Data Sharing Statement

Olivieri-Mui. Antiretroviral Therapy Changes for Medicare Beneficiaries With HIV Transitioning to Long-Term Care. *JAMA Netw Open*. Published December 12, 2025. doi:10.1001/jamanetworkopen.2025.48936

Data
Data available: No

Additional Information
Explanation for why data not available: These data are owned and access governed by the Centers for Medicare and Medicaid Services through a fee-based system precluding sharing of any data.
